# Supplementary material for: DNA Footprints: Using Parasites to Detect Elusive Animals, Proof of Principle in Hedgehogs
Source: Animals (Basel). 2020 Aug 14;10(8):1420. doi: 10.3390/ani10081420 (PMC7460031; doi:10.3390/ani10081420)
Supplement: Supplementary file 1 [file animals-10-01420-s001.pdf]

animals

Supplementary Materials: DNA Footprints: Using Parasites to Detect Elusive Animals, Proof of Principle in Hedgehogs

Simon Allen [1,2,*], Carolyn Greig [3], Ben Rowson [4], Robin B. Gasser [5], Abdul Jabbar [5], Simone Morelli [6], Eric R. Morgan [2,7], Martyn Wood [1] and Dan Forman [3]

1. Gower Bird Hospital, Sandy Lane, Parkmill, Gower, Swansea SA3 2EW, UK; martyn@gowerbirdhospital.org.uk
2. School of Biological Sciences, University of Bristol, Bristol Life Sciences Building, 24, Tyndall Avenue, Bristol BS8 1TQ, UK; eric.morgan@qub.ac.uk
3. College of Science, Swansea University, Singleton Park, Swansea, Wales SA2 8PP, UK; c.greig@swansea.ac.uk (C.G.); d.w.forman@swansea.ac.uk (D.F.)
4. Department of Natural Sciences, National Museum of Wales, Cardiff, Wales CF10 3NP, UK; ben.rowson@museumwales.ac.uk
5. Faculty of Veterinary and Agricultural Sciences, The University of Melbourne, Parkville, VIC 3010, Australia; robinbg@unimelb.edu.au (R.B.G.); jabbara@unimelb.edu.au (A.J.)
6. Faculty of Veterinary Medicine, Teaching Veterinary Hospital, University of Teramo, 64100 Teramo, TE, Italy; smorelli@unite.it
7. School of Biological Sciences, Queen's University Belfast, 19 Chlorine Gardens, Belfast, Northern Ireland BT9 5DL, UK

* Correspondence: simon@gowerbirdhospital.org.uk ; Tel.: +44-1792371630

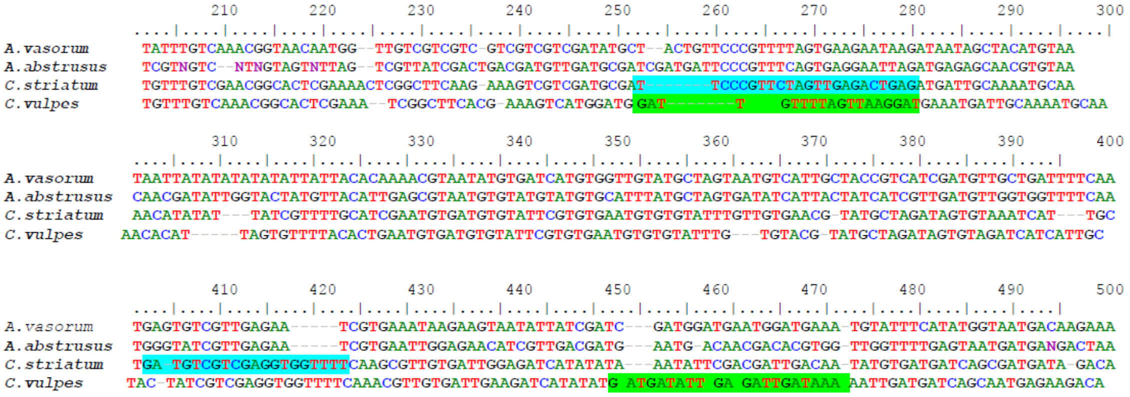

Figure S1. Sequence alignment of ITS-2 sequences showing positions of discriminatory primers resulting in specific PCR products differing in length by 50bp.

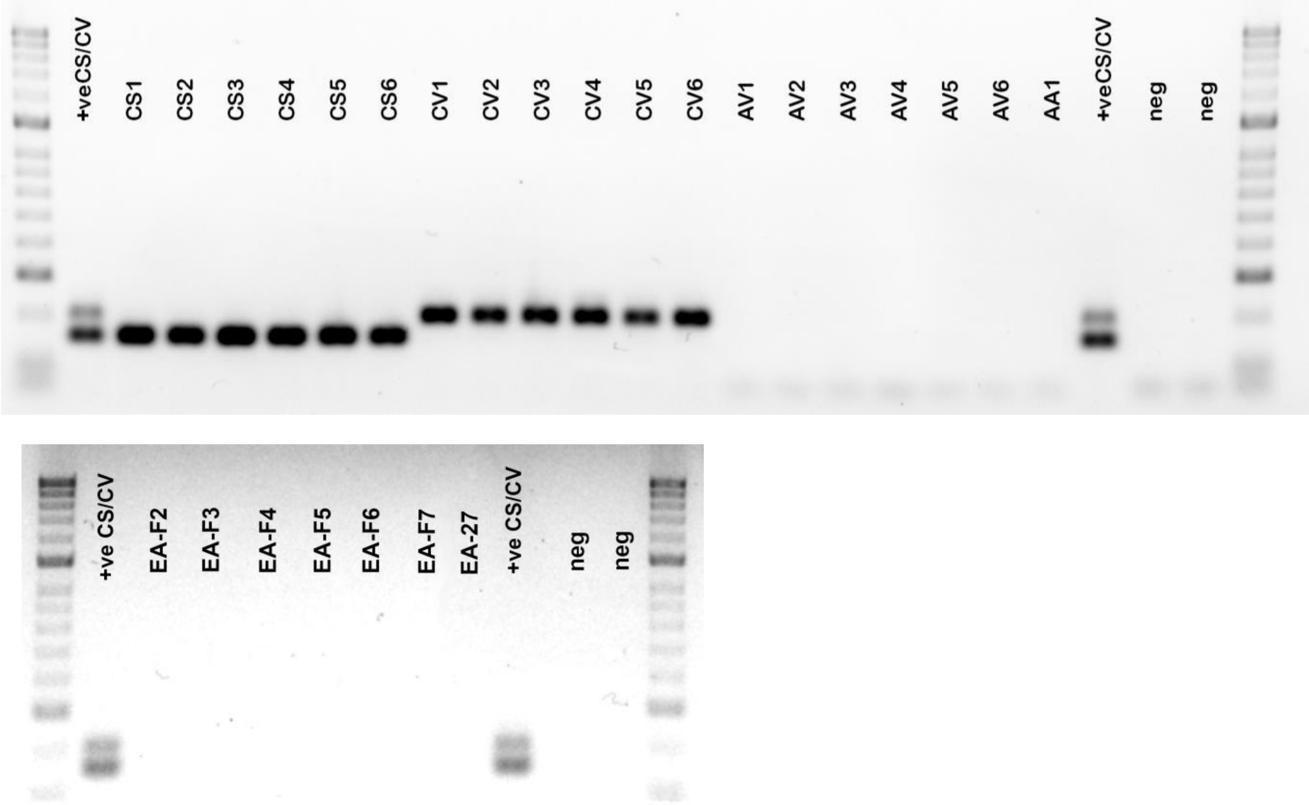

Figure S2. PCR of extracted nematode DNA with CS/CV multiplexed primer set illustrating specific amplification.

PCRs produced expected bands: 157 bp for *C. striatum* (CS), 207 bp for *C. vulpis (CV)* and no product amplified for other nematode species tested. **Gel 1** shows PCR of DNA extracts from *Crenosoma striatum* (CS) (hedgehog) *Crenosoma vulpis* (CV) (fox), *Angiostrongylus vasorum* (AV) (fox), and *Aelurostrongylus abstrusus* (AA) (cat) (kindly donated by S. Morelli). **Gel 2** shows PCR of DNA extracts from *Eucoleus aerophilus* (*Capillaria aerophila*) (EA) (fox). PCRs included a positive control mixture of DNA from *Crenosoma striatum* and *Crenosoma vulpis* and negative $H_2 0$ controls. All DNA was isolated from morphologically identified adult lungworms and validated for PCR with universal primers before testing.
